# Sensory Attenuation for Jointly Produced Action Effects

**DOI:** 10.3389/fpsyg.2013.00172

**Published:** 2013-04-11

**Authors:** Janeen D. Loehr

**Affiliations:** ^1^Department of Psychology, University of SaskatchewanSaskatoon, SK, Canada

**Keywords:** sensory attenuation, joint action, auditory N1 event-related potential, self-other distinction, social context

## Abstract

Successful joint action often requires people to distinguish between their own and others’ contributions to a shared goal. One mechanism that is thought to underlie a self-other distinction is sensory attenuation, whereby the sensory consequences of one’s own actions are reduced compared to other sensory events. Previous research has shown that the auditory N1 event-related potential (ERP) response is reduced for self-generated compared to externally generated tones. The current study examined whether attenuation also occurs for jointly generated tones, which require two people to coordinate their actions to produce a single tone. ERP responses were measured when participants generated tones alone (tone onset immediately followed the participant’s button press) or with a partner (tone onset immediately followed the participant’s or the partner’s button press, whichever occurred second). N1 attenuation was smaller for jointly generated tones compared to self-generated tones. For jointly generated tones, greater delays between the participant’s and the partner’s button presses were associated with reduced attenuation; moreover, only trials in which there was no delay between the participant’s press and tone onset showed attenuation, whereas trials in which there were delays did not show attenuation. These findings indicate that people differentiate between their own and another person’s contributions to a joint action at the sensorimotor level, even when they must act together to produce a single, shared effect.

## Introduction

Successful ensemble music performance relies on performers’ ability to take their co-performers’ actions into account when planning, executing, and monitoring their own actions. For instance, when a pianist and violinist play a duet together, each must plan and execute actions that complement and coincide with the other’s, each must adjust to the anticipated and perceived actions of the other, and each must monitor the consequences of their own and their partner’s actions to ensure that both individual and shared goals are met (Loehr et al., 2013). At the same time, however, each performer must maintain a distinction between their own and the other person’s actions. Only their own actions can be adjusted in response to the other’s actions; only their own errors can be corrected when individual or shared goals are not met. Although a substantial body of research has investigated the mechanisms that allow people to take each other’s actions into account during joint actions such as music performance (see Knoblich et al., 2011, for a review), less research has investigated the mechanisms that allow people to maintain a self-other distinction in joint action contexts. The current study examines whether one proposed mechanism for this distinction, sensory attenuation, allows people to distinguish between their own and others’ actions when they must coordinate them to produce a shared outcome.

People’s ability to take others’ actions into account during joint action relies in part on their ability to map others’ actions onto their own motor repertoires (Bekkering et al., 2009; Knoblich et al., 2011). Much of the evidence for the proposition that people represent their own and others’ actions using the same neural resources comes from studies in which one person observes another’s actions. Similar brain regions are activated when people execute an action and when they observe another person performing the action (Rizzolatti and Craighero, 2004; Rizzolatti and Sinigaglia, 2010) or predict that another person will perform the action (Kilner et al., 2004). Likewise, similar neural mechanisms are involved in detecting observed errors and detecting one’s own errors (van Schie et al., 2004). Shared representations of one’s own and others’ actions also support joint action (Bekkering et al., 2009; Knoblich et al., 2011). For example, the same anticipatory motor activity that precedes one’s own action is evident when one anticipates an interaction partner’s action (Kourtis et al., 2010). In joint music performance, unexpected feedback (pitches) in a partner’s part of a duet elicits the same neural action-monitoring processes as unexpected feedback in the performer’s own part of the duet (Loehr et al., in press). Studies of joint music performance have also yielded behavioral evidence that people use their own motor systems to simulate the actions of their duet partners, which facilitates temporal coordination (Keller et al., 2007; Loehr and Palmer, 2011).

Evidence that people represent their own and others’ actions using the same neural resources has raised the question of how people nevertheless maintain a distinction between their own and others’ actions (Decety and Sommerville, 2003; Jeannerod, 2003). One cue that has been proposed to underlie the self-other distinction is the suppression of self-generated sensorimotor signals (Miall and Wolpert, 1996; Schütz-Bosbach et al., 2009). Consistent with this hypothesis, motor-related activity associated with the left-hand accompaniment to a right hand melody is suppressed when pianists imagine performing the accompaniment themselves while they play the melody. In contrast, motor-related activity associated with the left-hand accompaniment is facilitated when pianists hear or imagine a duet partner performing it (Novembre et al., 2012). Suppressed and facilitated corticospinal excitability have also been associated with self- and other-related motor representations, respectively, in non-musical tasks (Schütz-Bosbach et al., 2006). Suppression or attenuation of the *sensory consequences* of self-generated actions is likewise thought to underlie the self-other distinction (Frith et al., 2000). Sensory attenuation is thought to occur when the sensory consequence of an action matches the prediction of a forward model that simulates how the body and environment will respond to an outgoing motor command (Miall and Wolpert, 1996). Self-generated sensory consequences are perceived as less intense and elicit reduced neural responses compared to externally generated sensory effects in somatosensory, auditory, and visual domains (see Waszak et al., 2012, for a review).

Most investigations of sensory attenuation have compared responses to self-generated and computer-generated sensory effects (Hughes et al., 2013). In the auditory domain, for example, tones that result from one’s own movements are perceived as less loud than tones produced by a computer (e.g., Weiss et al., 2011a). Similarly, the auditory N1, a negative-going event-related potential (ERP) component that peaks approximately 100 ms after the onset of a tone, is reduced for self-generated compared to computer-generated tones (e.g., Schafer and Marcus, 1973; Lange, 2011). Researchers have just begun to directly investigate whether sensory attenuation differentiates between sensory effects produced by oneself compared to another person. One early study showed that tones produced by oneself and tones produced by an observed other person were both attenuated relative to computer-generated tones (Sato, 2008). However, subsequent studies using a similar paradigm have shown that attenuation occurs for self-generated tones but not for other-generated tones (Weiss et al., 2011a; Weiss and Schütz-Bosbach, 2012). The main difference between these studies is that in the original (Sato, 2008), similarity between the participant and the observed other was maximized; the two wore identical gloves, pressed the same button, and produced the same tone. In subsequent studies, the observed other did not wear the same glove or press the same button as the participant, although both produced the same pitch in some cases. These findings suggest that similarity between self and other may influence whether a self-other distinction occurs at the sensorimotor level.

Other researchers have examined sensory attenuation under conditions in which the agent of the tone (oneself or another person) is ambiguous. Desantis et al. (2012) had people produce button presses in response to a visual stimulus. They were led to believe, *via* properties of the visual stimulus, that the tones they heard either resulted from their own button presses or from the button press of an experimenter who was hidden by an occluder. In reality, all tones were produced by the participants. Tones that participants believed to be self-generated were perceived as less loud than tones that participants believed to be experimenter-generated. Thus, a self-other distinction occurred at the sensorimotor level based solely on participants’ beliefs about who was the agent of the sensory effect.

Finally, one study has investigated attenuation as the basis for a self-other distinction in a social setting. Weiss et al. (2011b) compared attenuation for self- and other-generated tones in a typical solo action setting, in which participants produced tones or observed another person producing tones alone, to attenuation in a social setting, in which each person produced tones at the request of the other (signaled by an arm touch). Consistent with most previous research, self-generated tones were perceived as less loud compared to other-generated tones in both settings. However, self-generated tones were more attenuated in the social setting than in the solo setting, suggesting that self-related sensory signals may be enhanced when actions are performed in a social context. Furthermore, other-generated tones were also attenuated in the social setting, suggesting that another person’s actions may be incorporated into one’s own sensorimotor prediction loop in a social context.

Based on the findings just reviewed, it is unclear whether sensory attenuation can differentiate self from other in joint actions that require two people to coordinate their actions to achieve a shared goal. On one hand, self-related sensory signals may be enhanced in such a social context, preserving the attenuation-based self-other distinction; on the other hand, a partner’s actions may be incorporated into one’s sensorimotor prediction loop, leading to attenuation of the sensory consequences of both one’s own and one’s partner’s actions. The current study adapted the experimental paradigm used by Sato (2008) and subsequent investigations (Weiss et al., 2011a; Weiss and Schütz-Bosbach, 2012) to compare attenuation of self-generated tones, produced by a single participant acting alone, and jointly generated tones, produced by the participant acting together with a partner. Self-generated tones occurred immediately after the participant’s button press; jointly generated tones occurred only after both the participant and the partner had pressed their respective buttons. Attenuation was measured using the amplitude of the auditory N1 ERP, which has been shown to be reduced for self- compared to computer-generated tones in a number of studies (Waszak et al., 2012).

Two predictions can be made based on the existing literature. First, equivalent attenuation may occur for self and jointly generated tones (measured relative to computer-generated tones). This pattern would be consistent with Sato’s (2008) finding that equivalent attenuation for self- and other-generated tones occurs when the similarity between self and other is maximized. In the current study, similarity between self and other was high: although the participant and the partner pressed separate buttons, they did so (nearly) simultaneously to produce the same (single) tone. This pattern would also be consistent with Weiss et al.’s (2011b) finding that people may incorporate others’ actions into their sensorimotor prediction loops when they perform actions in a social setting; this process may be more likely to occur when people must coordinate their actions to achieve a single shared goal.

Second, attenuation may be reduced in the joint setting to the extent that delays occur between the participant’s and the partner’s actions (and hence, between the participant’s action and tone onset). This pattern would indicate that people use temporal cues to differentiate between their own and a partner’s contributions to producing a shared sensory effect. This pattern would be consistent with Desantis et al.’s (2012) finding that external cues (beliefs about who produced the tone) lead to self-other differences in attenuation when the agent of a sensory effect is ambiguous. Given that external cues are used to differentiate self- from other-generated effects, cues inherent to the sensorimotor signal itself may also be used to differentiate self- from other-generated effects. This pattern would also be consistent with Weiss et al.’s (2011b) finding that self-related sensory signals may be enhanced in a social setting. Finally, this pattern would be consistent with research showing that temporal cues are a crucial source of information for the self-other distinction at higher cognitive levels. For example, delays between a participant’s movement and its sensory consequences weaken people’s sense of agency (i.e., their sense of control over actions and their consequences; Sato and Yasuda, 2005).

## Materials and Methods

### Participants

Forty-eight adults (10 male, mean age = 22.71, SD = 3.88) participated in the study in pairs. Fourteen of the 24 pairs consisted of two females, and 10 pairs were mixed-gender. Five of the pairs knew each other before the experiment. EEG was measured from one randomly chosen member of each pair (henceforth referred to as *participants*; 3 male, 3 left-handed, mean age = 22.38, SD = 4.42). The other member of the pair served as the participant’s partner (referred to as *partners*), from whom only behavioral data were collected. All participants provided written informed consent according to procedures reviewed by the medical ethics committee at Radboud University Nijmegen. Participants were compensated with either course credit or €20 for their participation.

### Apparatus and materials

The experimental paradigm used by Sato (2008) was adapted to include a joint setting and to allow attenuation to be measured using ERPs. During the experiment, participants and partners sat next to each other on the same side of a table. The participant always sat on the right and the partner on the left. A computer screen was centered between them at a distance of approximately 80 cm from the edge of the table. The participant and the partner each had a Logitech Gamepad F310 game controller aligned with their right hand, approximately 20 cm from the edge of the table. The game controllers were modified to include pressure sensitive buttons (2 cm in diameter) that registered presses without providing auditory feedback. For conditions in which button presses triggered tones, the tones were presented *via* speakers placed on either side of the computer screen. Two sinusoidal tones of 1000 and 1500 Hz were used as stimuli. Each tone had a duration of 100 ms including 20 ms rise/fall and was presented with a sound pressure level of 70 dB. All stimuli were presented using Presentation software (Neurobehavioral Systems, Inc., Albany, CA, USA), which also recorded the participants’ and partners’ button presses.

### Design and procedure

**Figure 1:**
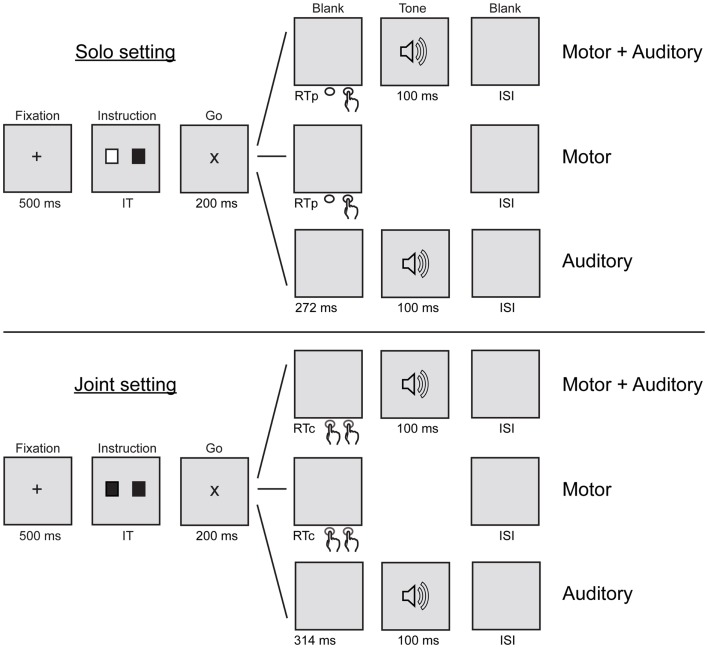
**Schematic illustration of the sequence of events in the motor + auditory, motor, and auditory tasks in the solo and joint settings**. IT, instruction time (1000, 1250, or 1500 ms); RTp, participant’s response time; RTc, coordinated response time (the longer of the participant’s or partner’s RT); ISI, inter-stimulus interval (0, 100, or 200 ms).

In order to measure sensory attenuation using ERPs, participants were asked to complete three tasks (motor + auditory, motor, and auditory) in each of the two settings (solo and joint). Figure 1 shows a schematic illustration of the six conditions resulting from the combination of tasks and settings. The solo setting is described first. In the solo motor + auditory condition, the participant pressed his or her button in order to produce a tone, which immediately followed the button press. In the solo motor condition, the participant pressed his or her button but no tone was produced. In the solo auditory condition, the participant listened to tones without pressing any buttons. In all three solo conditions, the partner sat quietly beside the participant. In the joint motor + auditory condition, the participant and the partner pressed their buttons together in order to produce a tone. Both people’s buttons had to be pressed before the tone would sound; thus the tone immediately followed the second of the two button presses. In the joint motor condition, the participant and the partner pressed their buttons together but no tone was produced. In the joint auditory condition, the participant (and partner) listened to tones without pressing any buttons. Trials in all six conditions followed the same visual cueing procedure described in the next paragraph. The motor conditions were included so that ERPs elicited by tone onsets that resulted from button presses in the motor + auditory conditions could be corrected for motor-related activity before being compared with ERPs elicited by tone onsets in the auditory conditions. The assignment of tones to conditions was counterbalanced across participants such that for half of the participants, the 1000 Hz tone was presented in the solo motor + auditory and auditory conditions and the 1500 Hz tone in the joint motor + auditory and auditory conditions, whereas the opposite was true for the other half of the participants.

Each trial consisted of the following sequence of events (see Figure 1). A white fixation cross was presented in the middle of a black computer screen. After 500 ms, the fixation cross was replaced by two squares presented 4 cm to the left and right of the center of the screen, respectively. The colors of the squares depended on the task of the participant (right square) and the partner (left square). The square was colored green if the participant or partner was required to press a button during the trial, and white if the participant or partner was not required to press a button during the trial. The squares remained on the screen for 1000, 1250, or 1500 ms (randomized across trials), followed by a “go” signal consisting of a white letter X. The go signal remained on the screen for 200 ms, after which a black screen was presented for up to 800 ms. Thus, participants (and partners) had up to 1000 ms to respond to the go signal. In the motor + auditory and motor conditions, participants (and partners) were instructed to press their buttons with the right index finger after the go signal appeared. In the motor + auditory conditions, tone onset occurred immediately after the participant’s button press (solo setting) or immediately after the participant’s or partner’s press, whichever occurred second (joint setting). In the auditory conditions, participants heard a tone after the go signal appeared. Tone onset time was equal to the average tone onset time over the last training block of the motor + auditory condition, calculated separately for the solo setting (*M* = 272.04 ms, SD = 46.81) and the joint setting (*M* = 313.98 ms, SD = 49.24). This ensured that tone onsets were equally predictable and had similar timing in the auditory and motor + auditory conditions within each setting. The next trial began 0, 100, or 200 ms (randomized across trials) after the trial was complete.

After being set up for the EEG recording, participants performed a training session in order to learn the relationship between actions and their consequences (i.e., specific tones). The training session consisted of 150 trials of the solo motor + auditory condition and 150 trials of the joint motor + auditory condition^1^. The order of the solo and joint settings was counterbalanced across pairs. Participants then completed the test phase of the experiment, which consisted of all six conditions presented in separate blocks. The order of the three tasks (motor + auditory, motor, auditory) was counterbalanced across participants. Each task was performed in both the solo and joint settings before moving on to the next task. Solo and joint settings occurred in the same order as during the training trials. Each block contained 40 trials, and the set of six blocks was repeated three times so that participants performed 120 trials of each condition. Participants were instructed about which task they would perform at the beginning of each block.

### Data acquisition

EEG was recorded continuously from each participant using 32 active electrodes (Acticap, Brain Products GmbH, Germany), arranged according to an extended version of the 10–20 system at F7, F3, Fz, F4, F8, FC5, FC1, FCz, FC2, FC6, T7, C3, Cz, C4, T8, CP5, CP1, CP2, CP6, P7, P3, Pz, P4, P8, O1, Oz, and O2, using carefully positioned nylon caps. All electrodes were referenced to the left mastoid during recording. Vertical eye movements were monitored using pairs of bipolar electrooculography (EOG) electrodes positioned directly above and beneath the right eye, and horizontal eye movements were monitored using pairs of bipolar EOG electrodes positioned at the outer canthi of the eyes. Impedance was kept below 10 kΩ. EEG and EOG signals were amplified within a bandwidth of 0.05–100 Hz and digitized with a sampling frequency of 1000 Hz.

### Data processing and analysis

EEG data processing was performed off-line using Brain Vision Analyzer software (V. 1.05, Brain Products GmbH, Germany). EEG data were first re-referenced to the mean of both mastoid electrodes. Automated ocular correction was performed using the procedure by Gratton et al. (1983) to eliminate artifacts induced by horizontal or vertical eye movements. The data were filtered using a high-pass filter of 0.01 Hz (24 dB/oct) and a low-pass filter of 40 Hz (24 dB/oct) in order to remove slow drifts and excessive noise, respectively. The corrected EEG data were then segmented into epochs from 50 ms before to 300 ms after tone onset (in the motor + auditory and auditory conditions) or button press (in the motor conditions). Epochs were time-locked to the participant’s button press in the solo motor condition and to either the participant’s or the partner’s button press, whichever occurred second, in the joint motor condition. This ensured that the motor-related activity captured by the motor condition ERPs was equivalent to the motor-related activity captured by the motor + auditory condition ERPs, in which tone onsets occurred simultaneously (within 2 ms) with the participant’s button press (solo motor + auditory condition) or the second of the participant’s and partner’s button presses (joint motor + auditory condition). Individual trials were removed if they contained further artifacts induced by head, body, or arm movements, as indicated by a difference between the maximum and the minimum value within a given segment that exceeded 125 μV. Individual trials were also excluded from analysis if the participant or the partner failed to press their buttons within a 1000 ms window following the go signal, or if the participant’s or the partner’s response time exceeded 2.5 standard deviations above their mean response times. These errors occurred in 11% of all recorded trials, typically because the participant or partner failed to press the pressure sensitive button hard enough to register a press. Average ERP waveforms were calculated separately for each subject and condition. The baseline period was set from 50 ms before to 50 ms after tone onsets/button presses to minimize misalignments of the waveforms due to anticipatory neural activity that may arise in a visual cueing paradigm (cf. Lange, 2011).

**Figure 2:**
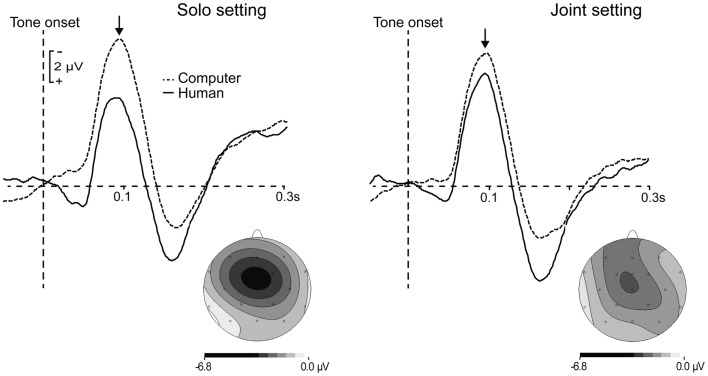
**Grand average waveforms time-locked to the onset of computer-generated (dashed lines) and human-generated (solid lines) tones in the solo and joint settings**. ERP responses to human-generated tones were corrected for motor activity related to button presses. Topographies show the scalp voltage distributions of the difference between computer- and human-generated tones in each setting. Arrows indicate the auditory N1 component.

Event-related potentials time-locked to tone onsets in the motor + auditory conditions were corrected for motor activity resulting from the button press required to produce the tone, consistent with previous analyses of the auditory N1 response to self-generated tones (Schafer and Marcus, 1973; Baess et al., 2008; Lange, 2011). This correction was accomplished by subtracting the ERPs time-locked to button presses in the motor condition from ERPs time-locked to tone onsets (which were simultaneous with either the participant’s or the partner’s button presses, as described above) in the motor + auditory condition. The correction was done separately for the solo and joint settings. In the figures and analyses that follow, motor-corrected ERPs from the motor + auditory conditions are referred to as ERPs elicited by *human-generated tones*, and are compared to ERPs elicited by tone onsets in the auditory conditions, referred to as *computer-generated tones*. Consistent with previous findings, the auditory N1 had a central scalp distribution, and the difference between ERPs elicited by human- and computer-generated tones was focal over electrode Cz (see Figure 2). The N1 was therefore defined as the mean amplitude at electrode Cz from 75 to 115 ms after tone onset, and was calculated separately for each participant and condition. The time window for this analysis was chosen based on the average peak latency of the N1 component elicited by human- and computer-generated tones in the solo and joint settings (*M* = 95.44 ms, SD = 10.07 ms), which did not differ across conditions (*F*s < 1.22, *p*s > 0.28).

The first analysis compared N1 amplitudes elicited by human- and computer-generated tones in the solo and joint settings using a repeated-measures ANOVA with factors agent (human, computer) and setting (solo, joint). Two further analyses focused on attenuation in the joint setting to determine whether people use temporal cues to distinguish between their own and others’ contributions to the jointly generated tones. The first analysis measured the correlation between sensory attenuation and asynchrony between participants’ and partners’ button presses, averaged over all trials in the joint setting. Sensory attenuation was calculated as the difference between the mean N1 amplitude elicited by computer-generated tones and the mean N1 amplitude elicited by jointly generated tones. Smaller values indicate less attenuation. Asynchronies were calculated as the onset of the participant’s button press minus the onset of the partner’s button press. Negative values indicate that the participant pressed before the partner, and hence a delay occurred between the participant’s button press and the tone onset. Thus, more negative asynchronies were expected to be associated with reduced attenuation if participants used temporal cues to differentiate between self and other.

The second analysis compared N1 amplitudes elicited by computer-generated tones to N1 amplitudes elicited by jointly generated tones whose onsets were delayed relative to the participant’s button press (i.e., trials in which the partner pressed after the participant, referred to as *delay* trials) and to N1 amplitudes elicited by jointly generated tones whose onsets occurred immediately after the participant’s button press (i.e., trials in which the participant pressed after the partner, referred to as *no-delay* trials). For this analysis, trials in the joint motor + auditory and joint motor conditions were divided into delay and no-delay trials. Trials were classified as no-delay if the asynchrony between the participant’s press and tone onset was no more than 10 ms (i.e., the asynchrony between the participant’s and the partner’s button press was greater than or equal to −10 ms). A cut-off value of 10 ms was used instead of a cut-off value of 0 ms to ensure that each participant had at least 10 trials of each type in each condition. Delays of 10 ms or less are unlikely to be perceptible to participants, as the just noticeable difference for tactile-auditory asynchronies is at least 20 ms (Harrar and Harris, 2008). To correct for motor-related activity, average ERP waveforms elicited in the motor condition were subtracted from average ERP waveforms elicited in the motor + auditory condition, separately for the delay and no-delay trials. Corrected ERPs in the delay and no-delay trials were then compared to ERPs elicited by computer-generated tones using a one-way repeated-measures ANOVA. All follow-up *post hoc* tests were conducted using paired-samples *t*-tests.

## Results

### N1 attenuation in solo and joint settings

The first analysis compared the amplitudes of the auditory N1s elicited by human- and computer-generated tones in the solo and joint settings. Figure 2 shows the grand average ERP waveforms in the four conditions, as well as the scalp voltage distributions of the difference between human- and computer-generated tones in each setting. N1 amplitude was attenuated (smaller) for human-generated tones compared to computer-generated tones, *F*(1, 23) = 10.95, *p* = 0.003. However, this was qualified by a significant interaction, *F*(1, 23) = 6.33, *p* = 0.02. *Post hoc* tests indicated that the difference in mean amplitude between human- and computer-generated tones was significant in the solo setting, *t*(23) = 3.84, *p* < 0.001, but was only marginal in the joint setting, *t*(23) = 1.81, *p* = 0.08.This finding cannot be attributed to a difference in the mean N1 amplitude elicited by computer-generated tones in the solo vs. joint settings, as these did not differ significantly, *t*(23) = 1.23, *p* = 0.23. Rather, mean N1 amplitude was smaller for human-generated tones in the solo setting compared to the joint setting, *t*(23) = 3.02, *p* = 0.006.

### Relationship between N1 attenuation and asynchronies in the joint setting

**Figure 3:**
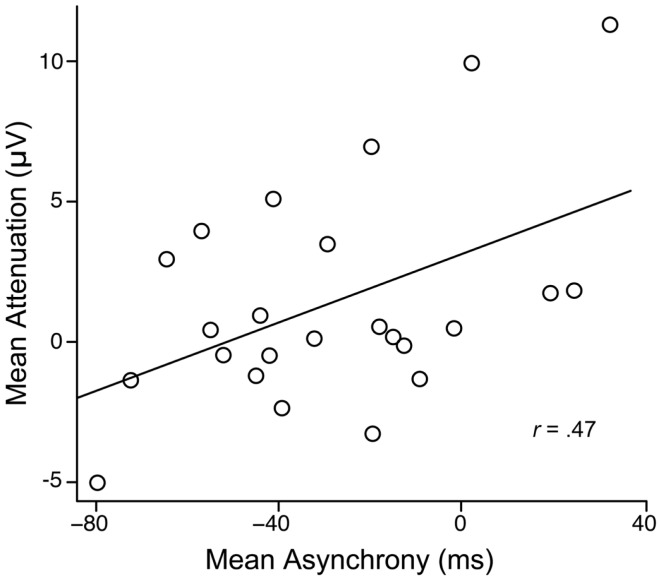
**Mean attenuation as a function of the mean asynchrony between participants’ and partners’ button presses in the joint setting**.

The average asynchrony between the participant’s and the partner’s button presses in the joint setting was −25.96 ms (SD = 32.02) and did not differ between the motor + auditory and motor tasks, *F*(1, 23) = 1.16, *p* = 0.29. Thus, on average, participants pressed their buttons before their partners did^2^. As shown in Figure 3, more negative asynchronies were associated with reduced attenuation, *r*(22) = 0.47, *p* = 0.02, indicating that longer delays between the participant’s button press and his or her partner’s button press (and hence, tone onset) led to reduced attenuation.

### N1 attenuation for delay and no-delay trials in the joint setting

60.17% percent of the motor + auditory trials and 60.38% of the motor trials were *delay* trials in which the participant’s button press occurred before their partner’s button press and there was therefore a delay between the participant’s press and the tone onset (*M*_motor+auditory_ = 62.13 delay trials per participant, range = 20–93; *M*_motor_ = 61.88, range = 17–94). The remaining trials were *no-delay* trials, in which participants’  button presses occurred after their partner’s and there was no delay between the participant’s press and the tone onset (*M*_motor+auditory_ = 41.67 no-delay trials per participant, range = 12–93; *M*_motor_ = 40.63, range = 11–89).

**Figure 4:**
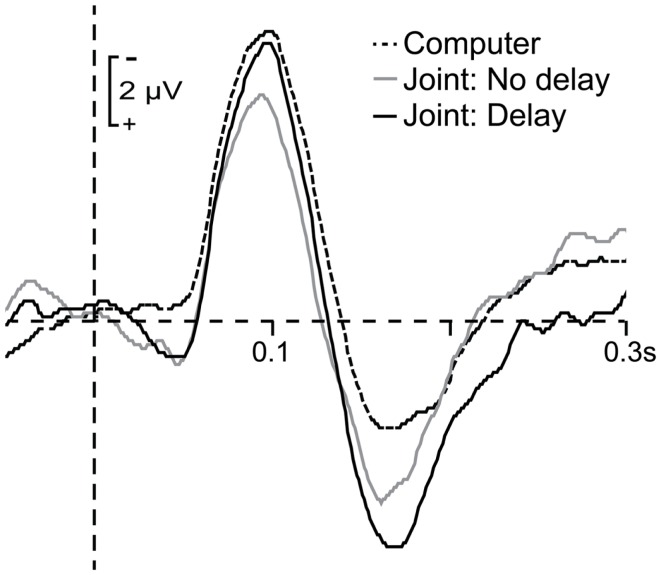
**Grand average waveforms time-locked to the onset of computer-generated tones (dashed line), jointly generated tones in no-delay trials (solid gray line), and jointly generated tones in delay trials (solid black line)**. ERP responses to jointly generated tones were corrected for motor activity related to button presses.

Figure 4 shows the average ERPs elicited by jointly generated tones in the delay and no-delay trials as well as the average ERP elicited by computer-generated tones in the joint setting. As the figure shows, the mean amplitude of the auditory N1 was only reduced in the joint setting when there was no delay between the participant’s press and tone onset. This was confirmed by a significant one-way ANOVA, *F*(2, 46) = 3.35, *p* = 0.044, and *post hoc* tests indicating that the difference between mean amplitudes elicited by computer-generated and no-delay tones was significant, *t*(23) = 2.51, *p* = 0.02, whereas the difference between mean amplitudes elicited by computer-generated and delayed tones was not significant, *t*(23) = 0.84, *p* = 0.41. Furthermore, the mean N1 amplitude elicited by no-delay tones in the joint setting did not differ significantly from the mean N1 amplitude elicited by human-generated tones in the solo setting, *t*(23) = 1.29, *p* = 0.21, indicating that attenuation of no-delay tones in the joint setting was of similar magnitude to attenuation of self-generated tones in the solo setting.

## Discussion

The current study examined sensory attenuation as a mechanism for the self-other distinction in the context of a joint action that required two people to coordinate their actions to produce a single shared action effect. Sensory attenuation was measured for self-generated tones whose onsets occurred immediately after the participant produced an action alone and for jointly generated tones whose onsets occurred only after both the participant and a partner produced coordinated actions. Participants’ neural responses to self-generated tones, as measured by the auditory N1 ERP, were attenuated compared to computer-generated tones, consistent with previous research (Schafer and Marcus, 1973; Lange, 2011). Participants’ neural responses to jointly generated tones were only marginally attenuated compared to computer-generated tones. Reduced attenuation in the joint setting was associated with greater delays between the participant’s and the partner’s button presses. Moreover, only trials in which there was no delay between the participant’s press and tone onset showed attenuation, whereas trials in which there were delays did not show attenuation. Thus, the marginal attenuation evident in the joint setting can likely be attributed to trials with no delay (40% of all trials). Together, these findings indicate that people use temporal cues to differentiate between their own and others’ contributions to producing a shared sensory effect.

The current findings support the hypothesis that sensory attenuation underlies a self-other distinction under conditions in which the agent of a sensory effect is ambiguous. Previous research created ambiguity through an experimental context that led participants to believe that tones were either produced by themselves or by another person hidden behind an occluder (e.g., Desantis et al., 2012). The current study created ambiguity by requiring two people to perform coordinated actions in order to produce a single, shared sensory effect; thus, both people’s actions were necessary for the sensory effect to be produced. Nevertheless, sensory attenuation differentiated between participants’ and partners’ contributions to producing the shared effect; this differentiation was based on the only cue available in the sensorimotor signal, the temporal relationship between each person’s actions and the jointly produced sensory effect.

Previous work has investigated whether the temporal relationship between actions and their sensory consequences influences attenuation in solo action contexts, with mixed results. Although some studies have shown that attenuation is reduced when the timing of the sensory effect is unpredictable relative to the action that caused it (Baess et al., 2008), others have shown that attenuation is not affected by whether the timing of the sensory effect is predictable (Lange, 2011). Likewise, some research has shown that attenuation is reduced as the delay between an action and its consequence increases (Schafer and Marcus, 1973; Aliu et al., 2008, Experiment 1). However, other work has shown that the effect of temporal delay depends on the training context that precedes the trials on which attenuation is measured. Whereas most studies employ a training phase in which no delay occurs between actions and their consequences, Aliu et al. (2008, Experiment 3) showed that when participants were trained to expect tones at non-zero delays, sensory attenuation generalized to a variety of delays during subsequent trials. The training phase in the current experiment comprised the same timing as the test phase; participants were trained with zero delay in the solo setting and variable delays in the joint setting. Attenuation should therefore have occurred at variable delays in subsequent trials in the joint setting, but this was not the case. Instead, participants relied on the timing they were trained to expect in the solo setting (no delay between their solo action and its auditory consequence) to differentiate self from other in the joint setting. This is consistent with Weiss et al.’s (2011b) finding of enhanced self-related sensory processing in a social setting. However, further work is needed to directly compare the effect of temporal delays in solo and joint settings, in order to determine the degree to which social setting and temporal delays independently affect attenuation and the self-other distinction.

The current findings indicate that people use temporal cues to differentiate between their own and others’ contributions to a shared action effect at the sensorimotor level. This is consistent with research showing that temporal cues have a role in the self-other distinction at higher cognitive levels. Delays between actions and their consequences weaken people’s sense of agency or control over sensory effects (Sato and Yasuda, 2005). Temporal cues also allow people to differentiate between their own and others’ previously recorded actions (Flach et al., 2004; Repp and Knoblich, 2004) and between self- and external control over ongoing perceptual effects (Repp and Knoblich, 2007). The relationship between the self-other distinction measured at the sensorimotor level (attenuation) and at higher cognitive levels (explicit ratings of agency) is a matter of debate (Gentsch and Schütz-Bosbach, 2011; Kühn et al., 2011; Gentsch et al., 2012). Current theory suggests that distinctions at the sensorimotor level contribute to a pre-reflective “feeling of agency,” which is integrated with other cues such as beliefs or intentions to produce an explicit “judgment of agency,” on which agency ratings are based (Synofzik et al., 2008). The relationship between sensorimotor signals and people’s sense of agency is particularly interesting in joint action, in which people’s sense of (self-)agency may remain intact or may be blurred into a sense of collective “joint-agency” (Pacherie, 2012). Agency in the context of joint action has just begun to be explored (van der Wel et al., 2012); whether suppression at the sensorimotor level determines which type of agency is experienced in a joint action is an intriguing avenue for further work.

Another avenue for further work concerns the role of attenuation in maintaining a self-other distinction in the context of more complex joint actions, such as the duet music performance described in the Section “Introduction.” More complex joint actions typically contain multiple cues on which a self-other distinction can be based. For example, when people produce independent sequences of sounds that are coordinated in time as in ensemble music performance, the self-other distinction may be based on differences in the pitch, timbre, loudness, or timing of each person’s tones. Asking participants to work together to produce a single, shared tone allowed the influence of temporal cues to be isolated in the current study. Whether and how other cues influence the self-other distinction in more complex joint actions remains to be determined. However, the current findings are broadly consistent with (Novembre et al.’s 2012) findings that suppressed motor-related activity was associated with one’s own compared to another performer’s part of a piano duet. Moreover, the current findings are consistent with the hypothesis that suppression of the sensorimotor consequences of self-generated actions underlies the self-other distinction (Miall and Wolpert, 1996; Frith et al., 2000; Schütz-Bosbach et al., 2009), even in the context of coordinated joint action.

In conclusion, the current study comprises a first step toward examining sensory attenuation as a mechanism for the self-other distinction in the context of joint actions that require people to coordinate their actions to achieve a shared outcome. The findings contribute to ongoing investigations of how self-other distinctions are maintained despite considerable evidence that people represent their own and others’ actions using similar neural resources (Decety and Sommerville, 2003; Jeannerod, 2003; Rizzolatti and Sinigaglia, 2010; Knoblich et al., 2011). The current findings indicate that people differentiate between their own and another person’s contributions to producing a shared effect at the sensorimotor level, and that they do so based on the temporal cues available in the sensorimotor signal. Promising avenues for future work include investigating the relationship between sensory attenuation and people’s experience of agency in the context of joint action, and investigating the role of sensory attenuation in maintaining a self-other distinction in more complex joint actions.

## Conflict of Interest Statement

The authors declare that the research was conducted in the absence of any commercial or financial relationships that could be construed as a potential conflict of interest.

## Footnotes

^1^The partner also performed a training session consisting of 150 trials of a solo motor + auditory condition in which the partner pressed his or her button in order to produce a tone. This training session was performed to reduce the likelihood that the participant’s and the partner’s reactions times would differ due to different amounts of experience with the task. A 700 Hz tone was used for the partner’s training, which was performed over headphones while the participant was being set up for EEG recording.

^2^Participants may have reacted more quickly to the go signal than partners did because they performed more test trials (solo and joint settings) than the partners performed (joint setting only).
